# Modeling the effects of lipid peroxidation during ferroptosis on membrane properties

**DOI:** 10.1038/s41598-018-23408-0

**Published:** 2018-03-26

**Authors:** Eran Agmon, Jérôme Solon, Patricia Bassereau, Brent R. Stockwell

**Affiliations:** 1 Department of Biological Sciences, MC4846, Columbia University, 550 West 120th Street, New York, NY 10027 USA; 2 Department of Chemistry, MC4846, Columbia University, 550 West 120th Street, New York, NY 10027 USA; 3 Laboratoire Physico Chimie Curie, Institut Curie, PSL Research University, CNRS UMR168, 75005 Paris, France; 4 Sorbonne Université, 75005 Paris, France; 5 Center for Genomic Regulation, The Barcelona Institute of Science and Technology, Barcelona, Spain; 6 Universitat Pompeu Fabra, Barcelona, Spain

## Abstract

Ferroptosis is a form of regulated cell death characterized by the accumulation of lipid hydroperoxides. There has been significant research on the pathways leading to the accumulation of oxidized lipids, but the downstream effects and how lipid peroxides cause cell death during ferroptosis remain a major puzzle. We evaluated key features of ferroptosis in newly developed molecular dynamics models of lipid membranes to investigate the biophysical consequences of lipid peroxidation, and generated hypotheses about how lipid peroxides contribute to cell death during ferroptosis.

## Introduction

## Lipid Composition of Ferroptosis

**Figure 1:**
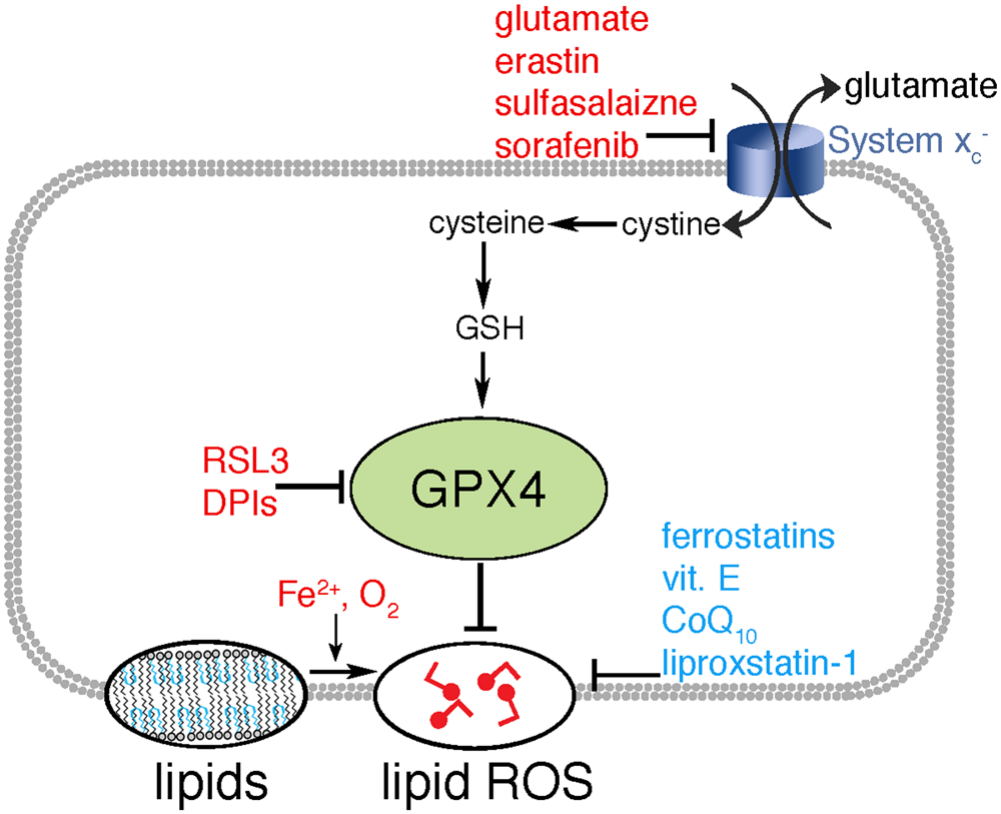
Inducers (red), and inhibitors (blue) of ferroptosis. GPX4 protects cells from lipid peroxidation; its inhibition by the depletion of GSH, or more directly through its binding with molecules such as RSL3, triggers accumulation of lipid oxygen reactive species (ROS), and trigger cell death.

Ferroptosis is distinct form of regulated cell death^1^, triggered ultimately by the loss of glutathione peroxidase 4 (GPX4) – a lipid repair enzyme – and results in the accumulation of lipid hydroperoxides, which ultimately cause cell death^2^. There has been much research devoted to discovering regulatory pathways leading to GPX4 inhibition (Fig. 1) and lipid peroxidation; we examined here the enigmatic process by which lipid peroxidation causes cell death, by simulating how lipid hydroperoxides influence membrane properties, and how these changes can contribute to the destruction of ferroptotic cells.

Numerous experiments have revealed the lipid compositions required for ferroptotic cell death: ferroptosis is characterized by the accumulation of oxidized polyunsaturated fatty acyl moieties (ox-PUFAs) within the context of phospholipid membranes. Synthesis of polyunsaturated fatty acids and their incorporation into phospholipid membranes is required for ferroptosis; in addition, the mevalonate pathway regulates sensitivity to ferroptosis through its downstream products CoQ_10_ and isopentenylated TRIT1, the latter being required for GPX4 production^3^. Acyl-CoA synthetase long-chain family member 4 (ACSL4) incorporates long polyunsaturated fatty acids (PUFAs) into membranes and is required for ferroptosis^4,5^. Lysophosphatidylcholine acyltransferase 3 (LPCAT3) inserts acyl groups into lysophospholipids (which have one fatty acyl tail), specifically towards the phospholipids phosphatidylcholine (PC) and phosphatidylethanolamine (PE), and is also required for cells to undergo ferroptosis^5,6^. Ferrostatins, liproxstatins, vitamin E and CoQ_10_ and their derivatives can inhibit the peroxidation of PUFAs in phospholipid membranes and thereby prevent ferroptosis.

**Figure 2:**
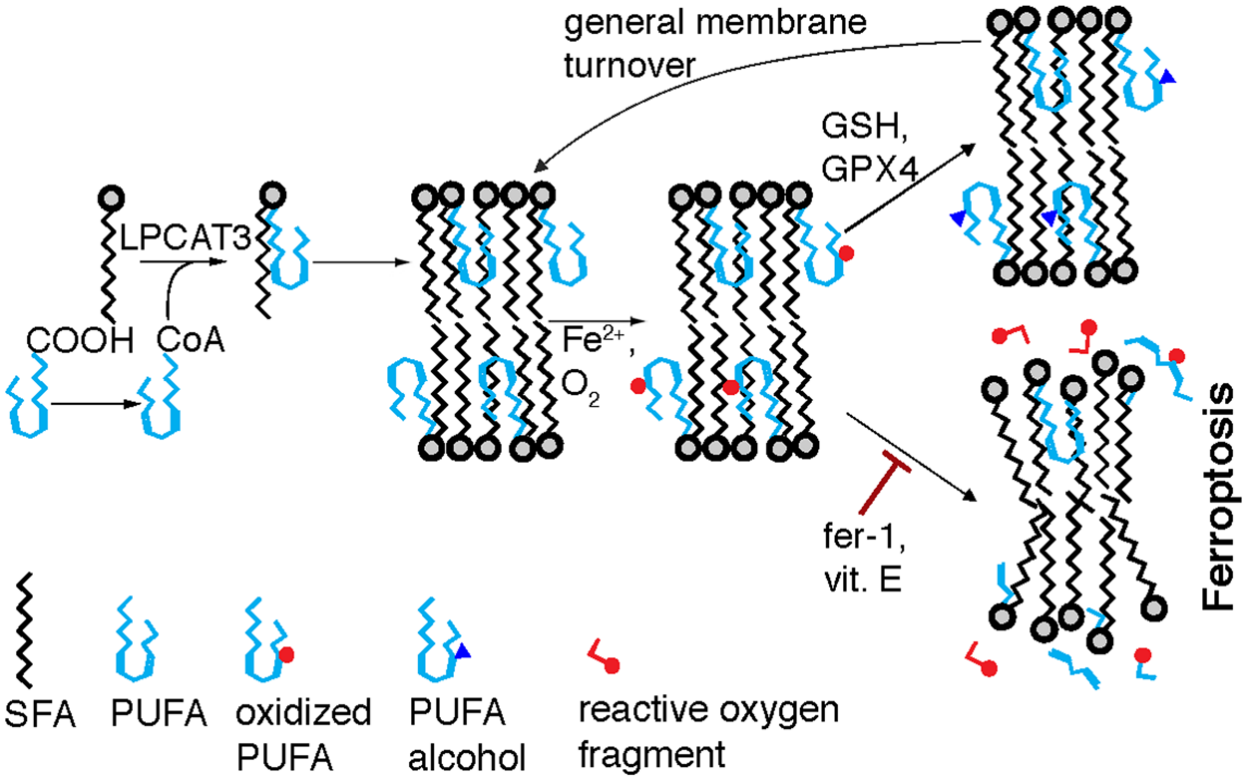
The role of lipids in ferroptosis. Polyunsaturated fatty acids (PUFAs), depicted in light blue, are acetylated by acyl-CoA synthetase and inserted into phospholipids with saturated fatty acids (SFAs). Ferroptosis involves oxidation (red circles) of PUFAs, which can be repaired with GPX4 by reducing the ox-PUFAs to lipid alcohols (blue triangles). In the absence of GPX4, ox-PUFAs can result the release of reactive oxygen species (red fragment) and membrane destruction.

There are many ways by which altered lipid composition could lead to cell death, but these possibilities are difficult to observe and evaluate directly. Plausible hypotheses for the connection between lipid peroxidation and ferroptotic cell death include that (1) compositional changes directly cause widespread membrane damage, as with the opening of pores leading to loss of ionic homeostasis, (2) local lipid domains are disrupted during lipid peroxidation, (3) compositional changes alter membrane-embedded proteins, interfering with their function, or (4) oxidized PUFA fragments are generated, releasing reactive species that disrupt other essential cellular processes or activate downstream lethal events. We examined here the plausibility of the simplest hypothesis that the change of lipid composition and its resulting membrane properties are themselves sufficient to cause cell death (Fig. 2).

**Figure 3:**
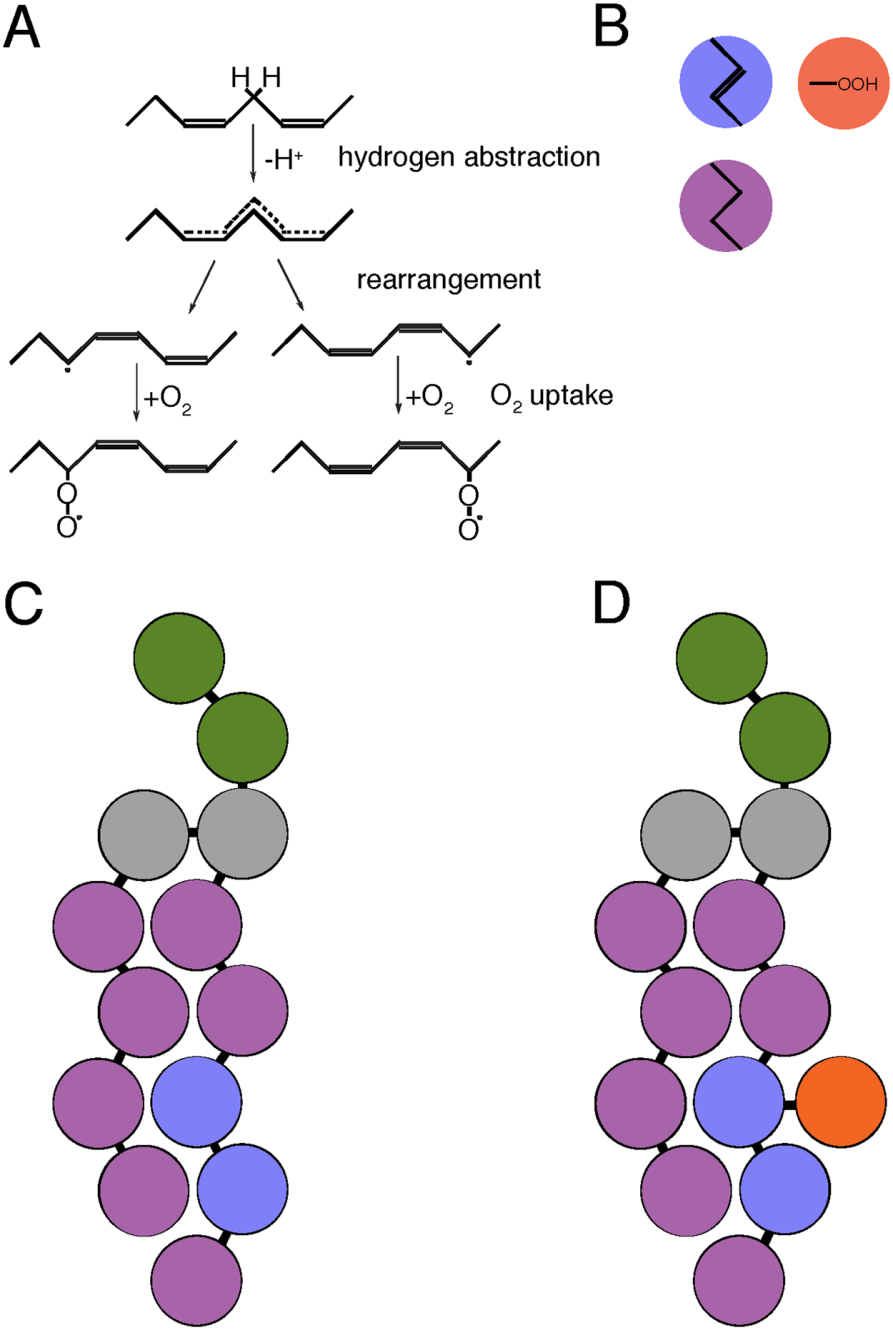
Peroxidation and coarse graining. (**A**) Peroxidation at double bond. This only happens at bis-allylic carbons. (**B**) Different MARTINI beads for three carbons with a double bond (blue), three saturated carbons (purple), and a peroxide (red). (**C**) A non-oxidized coarse-grained lipid. (**D**) An oxidized coarse-grained lipid.

We developed a molecular dynamics (MD) approach to study the properties of simulated membranes relevant to ferroptosis. MD is a computational method that simulates molecular movements by numerically solving Newton’s equations of motion for systems of many interacting atoms, or groups of atoms. At present, atomic-scale MD allows for modeling effects of ~5,000 lipids on the timescale of microseconds. To increase the spatial and temporal dimensions of MD simulations, however, there have been successful efforts to devise coarse-grained (CG) approximations that reduce models’ degrees of freedom. One of the most widely used CG methods is the MARTINI force field^7^, which gives realistic structure and properties of lipid bilayers. MARTINI runs on GROMACS, and has specialized in lipid simulations. A cartoon of the MARTINI coarse-graining method is shown in Fig. 3, with a special parameterized peroxide bead that we developed. The MARTINI force field has been used to model many lipid properties, including vesicle self-assembly, undulations of lipid bilayers, pore-formation, interactions with proteins, and lipid rafts^8^.

We used the MARTINI model with a newly parameterized peroxide bead, and simulated membranes with lipid compositions relevant to ferroptosis. These include membranes with increased PUFA concentrations, phosphatidylethanolamine (PE) head groups, long-tailed fatty acids, and peroxidized lipids. By evaluating the impact of compositional changes in lipids on membrane properties, the results revealed that compositional changes are sufficient to explain cell death resulting from ferroptosis. The changing shape and curvature of lipid membranes promotes increased accessibility to oxidants, which can expedite membrane destruction and bring about cell death.

Finally, we confirmed these computational results with an experiment using giant unilamellar vesicles (GUVs) to observe membrane changes following peroxidation. These GUVs were peroxidized by photobleaching and are observed to undergo membrane changes that are aligned with the model predictions, including membrane thinning and increase local membrane curvature. These results provide experimental confirmation of changing membrane properties during lipid peroxidation, and support the hypothesis that these changes occur during ferroptosis and could lead to cell death.

## Results

The set of experiments examined here consisted initially of scans of changing membrane compositions with different lipid species. The lipids were selected to highlight key features of the compositional changes relevant to ferroptosis. These include increased concentrations of PUFAs, increased concentrations of PEs, increased concentration of lipid peroxides, and longer acyl tails. The selected lipids are shown in Table 1: lipid species are abbreviated as four letters – the first and second designating the tail groups, and the third and fourth designating the head group. An “ox” before the lipid abbreviation means that the lipid has been oxidized. For the structure of the lipids, if there is a third number describing the lipid tail, it represents the number of peroxides on that tail (18:1:1 is an 18-carbon tail with 1 double bond and 1 peroxide).

**Table 1:** Lipid Species used in the coarse-grained MD model to examine the effects of ferroptosis.

Abbreviation	Name	Structure
DSPC	distearoyl-sn-glycero-3-phosphatidylcholine	18:0 PC
DOPC	dioleoyl-sn-glycero-3-phosphatidylcholine	18:1 PC
DLPC	dilinoleoyl-sn-glycero-3-phosphatidylcholine	18:2 PC
SOPC	1-stearoyl-2-oleoyl-sn-glycero-3-phosphatidylcholine	18:0–18:1 PC
SLPC	1-stearoyl-2-linoleoyl-sn-glycero-3-phosphatidylcholine	18:0–18:2 PC
DSPE	distearoyl-sn-glycero-3-phosphatidylethanolamine	18:0 PE
DOPE	dioleoyl-sn-glycero-3-phosphatidylethanolamine	18:1 PE
DLPE	dilinoleoyl-sn-glycero-3-phosphatidylethanolamine	18:2 PE
SLPE	1-stearoyl-2-linoleoyl-sn-glycero-3-phosphatidylethanolamine	18:0–18:2 PE
DHPE	didocosahexaenoic-sn-glycero-3-phosphatidylethanolamine	22:6 PE
oxDLPE	oxidized dilinoleoyl-sn-glycero-3-phosphatidylethanolamine	18:2:2 PE
oxSLPE	oxidized 1-stearoyl-2-linoleoyl-sn-glycero-3- phosphatidylethanolamine	18:0–18:2:2 PE
ox1DHPE	oxidized didocosahexaenoic-sn-glycero-3-phosphatidylethanolamine	22:6:2 PE
ox2DHPE	oxidized didocosahexaenoic-sn-glycero-3-phosphatidylethanolamine	22:6:4 PE

Three sets of experiments are shown in Table 2, each one highlighting key compositional changes seen in ferroptosis. These include (1) comparisons of different compositions of saturated fatty acids (SFA) and unsaturated/polyunsaturated fatty acids (UFA/PUFA), (2) comparisons of different compositions of PC and PE head groups, and (3) comparisons of different compositions of non-oxidized lipids and oxidized lipids. In each case, a typical lipid was incrementally replaced by a ferroptosis-relevant lipid, keeping all other aspects of lipid structures constant, while only changing the lipid structure of interest. An example is a comparison of distearoyl-sn-glycero-3-phosphatidylcholine (DSPC) and distearoyl-sn-glycero-3-phosphatidylethanolamine (DSPE) compositions, which is annotated as DSPC:DSPE. These lipids are alike in every respect except for their head group. A given scan started with a membrane composed entirely of one lipid, and several simulations were performed in which the proportion of the opposite lipid was increased until the membrane was entirely composed of the replacement lipid. Each scan was a set of 6 simulations – 6 different simulated membranes – starting with a membrane composed 100% of the starting lipid and moving at 20% increments towards 100% of the ferroptosis-relevant lipid. Each membrane simulation was repeated 10 times to examine reproducibility.

**Table 2:** MD Experiments consist of several membrane scans that highlight specific changes seen in the lipid composition of ferroptosis.

*Experiment*	*Scan*
SFA:PUFA	DSPC:DOPC
	DSPC:DLPC
	DSPC:SOPC
	DSPC:SLPC
PC:PE	DSPC:DSPE
	DOPC:DOPE
	DLPC:DLPE
Lipid:ox-lipid	DLPE:oxDLPE
	SLPE:oxSLPE
	DHPE:ox1DHPE
	DHPE:ox2DHPE

### Analysis of membrane properties

There are several ways to analyze properties of lipid bilayers in molecular dynamics simulations^9^. These include membrane bending energies, area per lipid and compressibility, bending and tilt of bilayers, permeability, and lipid diffusion. Of these, there were several properties that we found to change in these simulations, and which provide insight into the execution mechanism of ferroptosis. These include the accessibility to oxidants, curvature of the membrane, fluidity, permeability, micellization (the formation of micelles, or breaking off of micelles from a bilayer), and the formation of pores.

**Figure 4:**
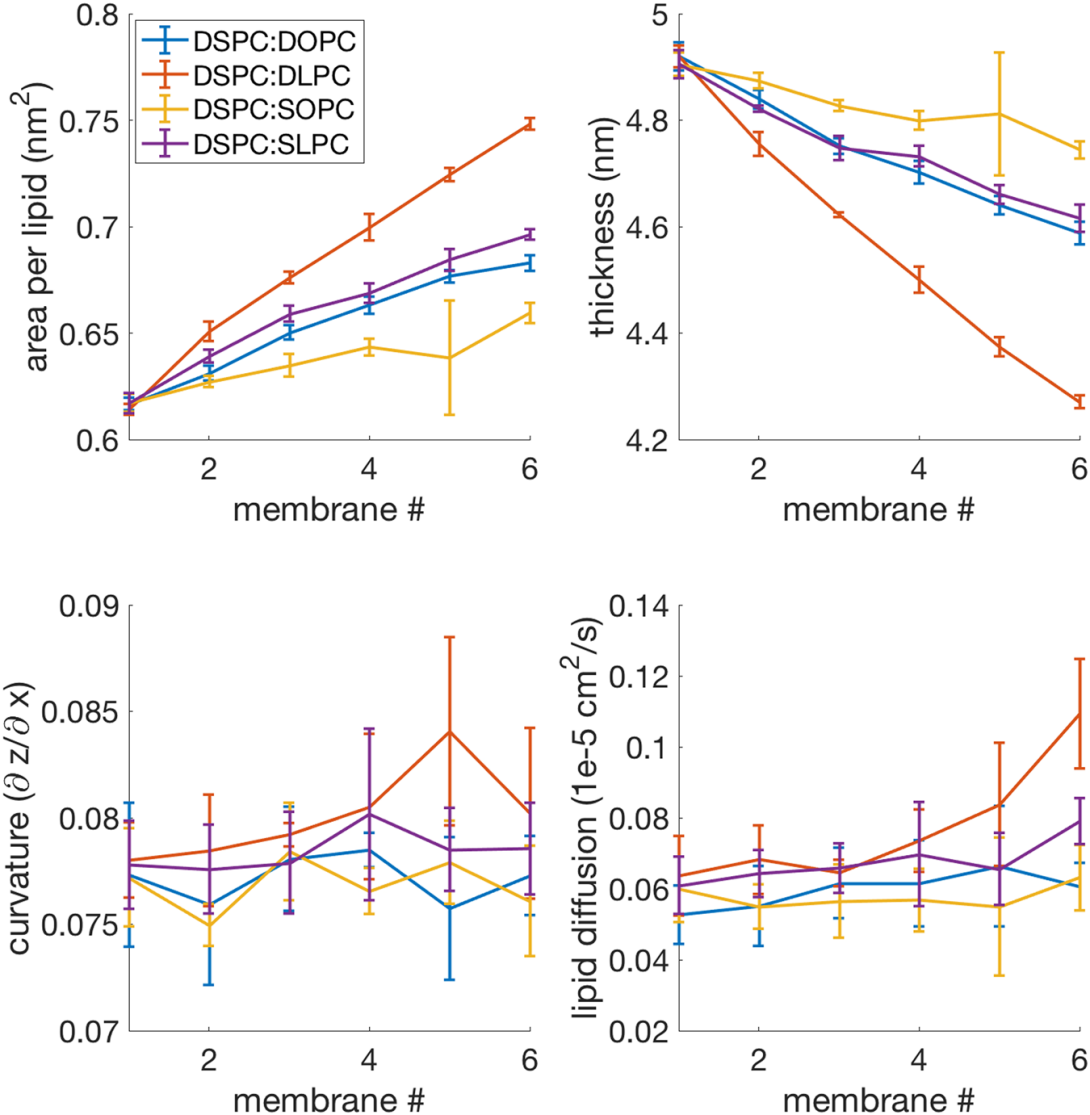
The SFA:PUFA experiment consists of 4 different scans; DSPC:DOPC, DSPC:DLPC, DSPC:SOPC, and DSPC:SLPC. Each one of these shows increasing area per lipid and decreasing thickness as it transitions towards a ferroptosis-relevant lipid. Lipid diffusion also increases slightly, with a more pronounced effect with DLPC.

**Figure 5:**
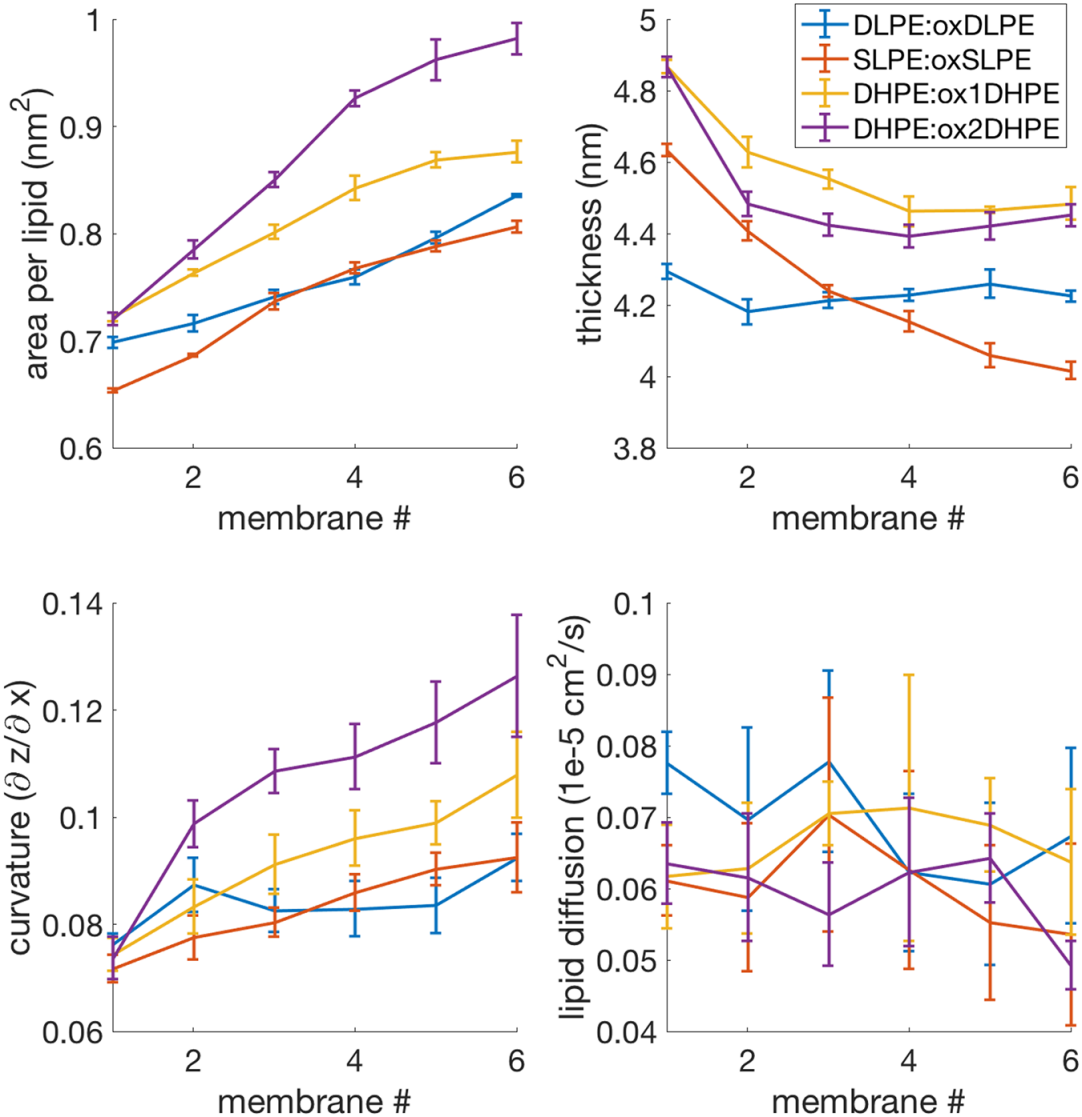
The lipid:ox-lipid experiment consists of 4 scans; DLPE:oxDLPE, SLPE:oxSLPE, DHPE:ox1DHPE, and the more highly oxidized DHPE:ox2DHPE. These reveal increased area per lipid, decreased thickness, and increased curvature.

**Figure 6:**
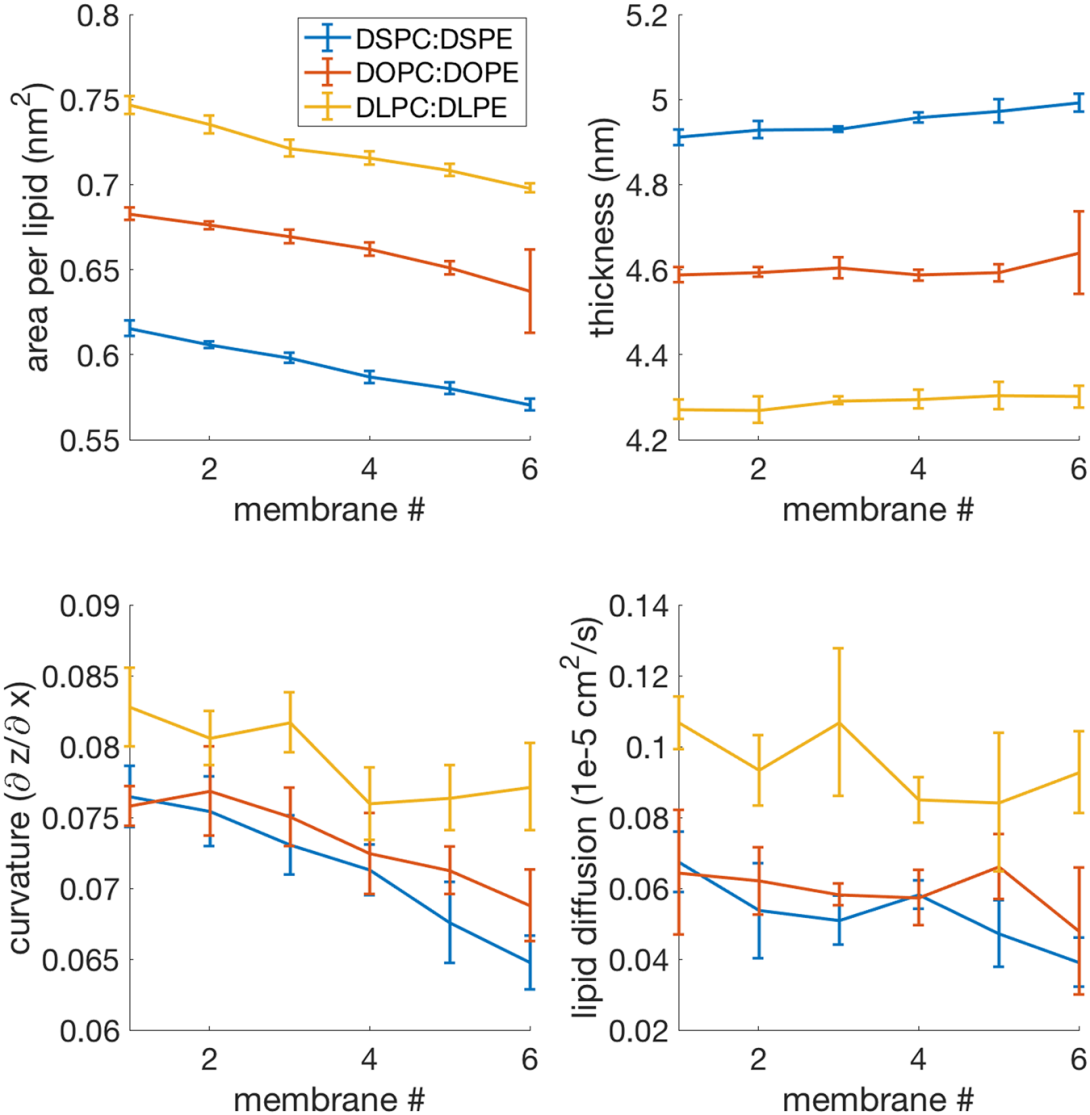
The PC:PE experiment consists of 3 scans; DSPC:DSPE, DOPC:DOPE, DLPC:DLPE. These reveal slight decreases in area per lipid, slight increases in thickness, curvature is reduced, and lipid diffusion remains constant.

Figures 4–6 examine four key properties that were observed to vary in the scans from non-ferroptotic to ferroptotic membranes. These properties include area per lipid, membrane width, curvature, and lipid diffusion. Area per lipid and membrane width together indicate accessibility to oxidants, with greater area per lipid and decreased membrane width having higher accessibility due to the more exposed lipid tails. Curvature indicates higher probability of micellization, even though micellization itself was not examined in these experiments, due to the timescale and size of the membranes used. Lipid diffusion represents the fluidity of the membrane, with higher diffusion allowing for proteins to move more freely through the membrane and increasing protein-protein interactions of membrane-embedded proteins – a decrease in this property indicates that protein-protein interactions would be suppressed.

The SFA:PUFA scans (saturated fatty acid to polyunsaturated fatty acids), shown in Fig. 4, revealed an increase in the area per lipid as more PUFAs were incorporated into membranes, as well as a decrease in membrane width. Lipid diffusion increased, with a more pronounced effect for DLPC, while curvature remained constant. This implied that the compositional change of increased PUFA incorporation allows for increased accessibility to oxidants, which may react with PUFAs inside of the membrane, further accelerating the drive to ferroptosis-competent membranes in a positive feedback loop.

The lipid-to-oxidized-lipid scans, shown in Fig. 5, similarly revealed an increase in the area per lipid, a decrease in the membrane width, and an increase in membrane curvature even more significantly, while lipid diffusion remained the same. These changes further contribute to increased accessibility of oxidants to the membrane interior, which should further drive peroxidation. The increase in curvature would also likely lead to micellization if taken to a larger spatial and temporal scale.

PC:PE scans (phosphatidylcholine head groups to phosphatidylethanolamine head groups, shown in Fig. 6, revealed a *decrease* in the area per lipid and a slight *increase* in membrane width, while curvature decreased and lipid diffusion remained constant. The presence of PE thus appears to counter the accessibility changes seen in the SFA:PUFA and non-oxidized lipid to oxidized lipid scans. PE might enable membrane destruction in other ways such as facilitating interactions between proteins and bilayer taking place due to PE’s small polar head, or it might serve as a buffering agent to prevent excessive ferroptosis until overwhelmed by the presence of lipid peroxides^10^. Moreover, the specific peroxidation of PE phospholipids during ferroptosis may serve to counter the buffering effect of PE on membrane curvature, area per lipid and membrane thickness, initiating the ferroptosis process at the sites that would otherwise be maintaining membrane integrity. This is analogous to removing the struts in a bridge during demolition.

**Figure 7:**
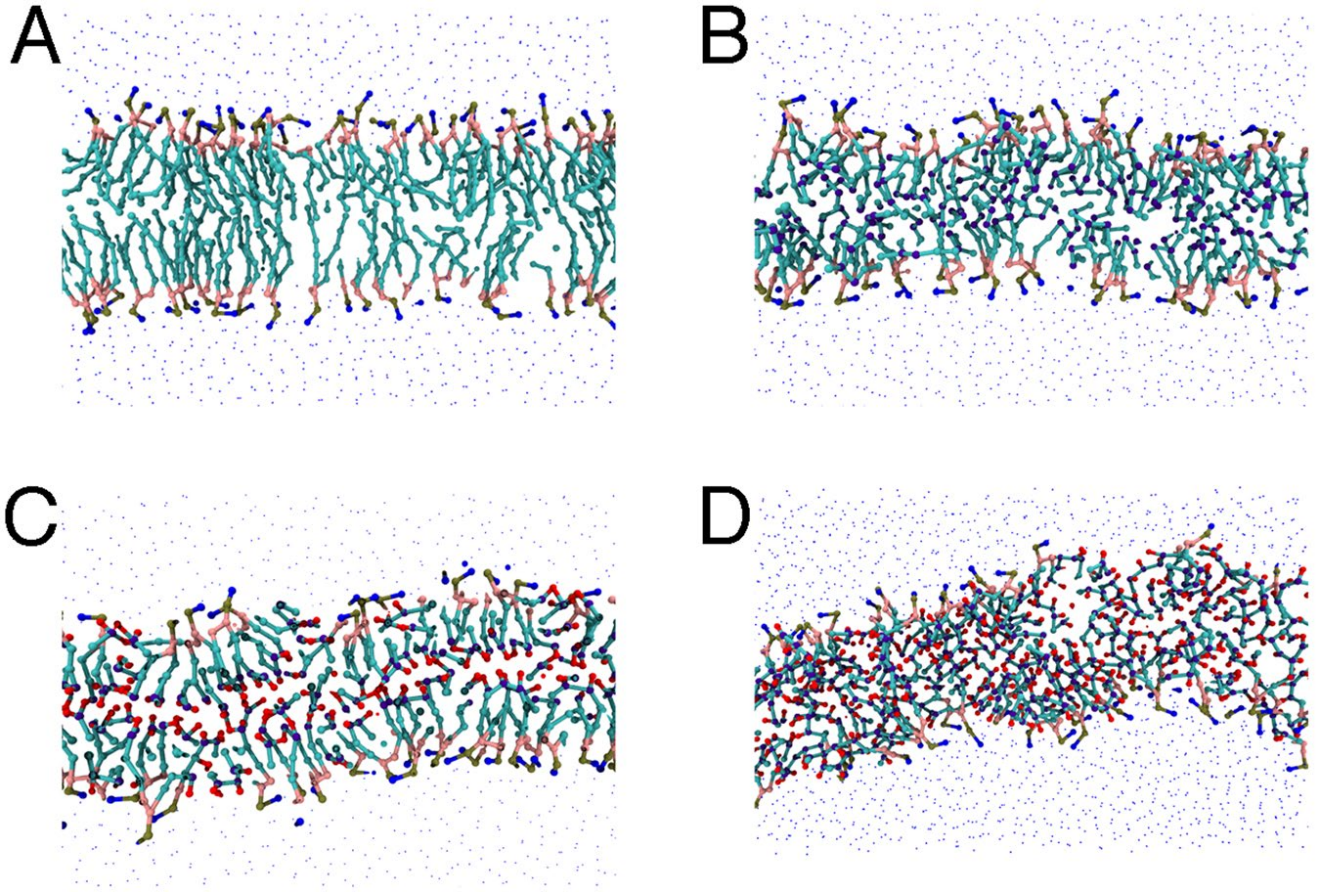
A cross-section of four membranes, highlighting the changes caused by ferroptosis. (**A**) A membrane composed of 100% DSPC. (**B**) A membrane composed of 100% oxidized DLPC. (**C**) A membrane composed of 100% oxDLPE. (**D**) A membrane composed of 100% ox2DHPE. Peroxide beads are shown in red, double-bond beads are shown in purple, saturated carbon beads are shown in cyan, glycerol linkers are pink, and the head groups are blue and yellow.

Figure 7 shows a cross section of four membranes, each composed of single lipid species. These show (A) the fully saturated DSPC, (B) the PUFA-tail DLPC, (C) the oxidized PUFA-tailed DLPE, and (D) the oxidized long-tailed ox2DHPE. These illustrate changes we observed when changing composition from SFA to PUFA, from unoxidized PUFA to oxidized PUFA, and from oxidized normal tails to oxidized long tails. The oxidized lipid tails bend towards the liquid phase, pushing aside the nearby lipids, thus increasing lipid area and decreasing width. Curvature is also very clearly increasing with the transition from DSPC to DLPC to oxDLPE to ox2DHPE. These changes increase the accessibly of oxidants into the bilayer because of the thinner packing of lipid head groups, and closer proximity of the lipid tails.

**Figure 8:**
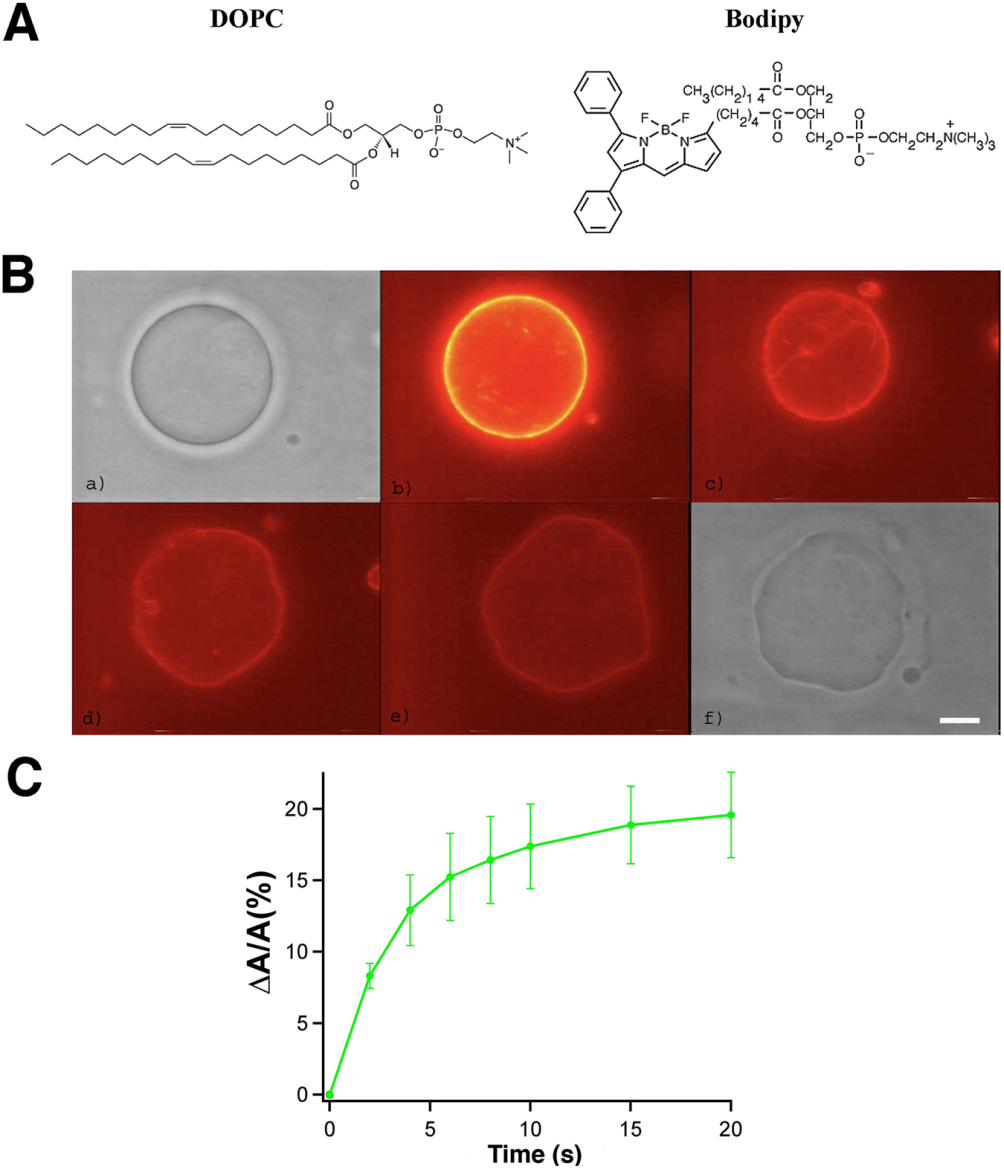
Experimental results demonstrating altered membrane properties following lipid peroxidation. (**A**) DOPC and fluorescent lipid C5-Bodipy-HPC. (**B**) A vesicle with 95% DOPC and 5% Bodipy. Upon illumination in panel (b), photobleaching begins and lipid peroxides form. Within 10 seconds, from panels (b) to (e), the membrane visibly fluctuates more and more and highly curved area appear. (a and f) The same GUV observed before and after illumination by phase contrast microscopy. Scale bar, 10 μm. (**C**) Relative area change versus time as measured with micropipette aspiration (N = 10). t = 0 corresponds to the start of the fluorescent illumination.

As a first step towards observing membrane changes during ferroptosis, we used model membranes in Giant Unilamellar Vesicles (GUVs) made of a mixture of 95% DOPC (i.e. with one unsaturation per chain) and 5% of the fluorescent lipid C5-Bodipy-HPC (Fig. 8A). The Bodipy 530–550 fluorophore is on the chain of the lipid and is thus embedded into the lipid bilayer of the GUV. Free radicals created upon photobleaching have a high probability of oxidizing the double bonds and forming lipid peroxides^11^. We observed by optical microscopy that a GUV with an initially smooth spherical shape (Fig. 8B–a), started to fluctuate immediately upon laser illumination and photobleaching (Fig. 8B–b) and developed local highly curved membrane area (Fig. 8B-c). The amplitude of the fluctuations and membrane instabilities increased over time over a typical time course of 10 seconds (Fig. 8B,c and e) and persisted when the illumination was stopped (Fig. 8B–f). In contrast, no shape change was observed even after ten minutes when the GUV was illuminated with white light via phase contrast, or when the fluorescent lipid was not added to the DOPC lipids (data not shown). Since the volume of the GUV remains a priori constant, the fluctuation enhancement upon photobleaching suggests that membrane tension is decreased, and thus that the total area of the GUV increases during the process. With micropipette aspiration, we quantified the area change upon photobleaching (Fig. 8C). We see that in about 20 seconds, the relative area has increased by about 20%. This area increase is in qualitative good agreement with our simulations, as well as with the thinning of the membrane they predict.

## Discussion

The MD simulations performed examined the effects of lipid peroxidation and other aspects of lipid composition relevant to ferroptosis on membrane properties. Increased ferroptosis-competent lipid compositions increase the accessibility of oxidants into the membrane interior and increase membrane curvature. As this occurs, oxidant accessibility and curvature would further exacerbate the situation, allowing more oxidation and therefore increased accessibility and curvature. In the absence of GPX4 being available to counter this process and remove lipid hydroperoxides, a runaway feedback cycle could arise that will ultimately destabilize the membrane, leading to pores and micellization. The fact that PE serves to counter this process and that ferroptosis lipid peroxidation specifically is targeted to PE phospholipids suggests that removal of the buffering effect of PE on lipid integrity may be a key aspect of ferroptosis. With this observation, it appears that lipid compositional change and resulting membrane properties might be sufficient to explain ferroptotic death, or at least represent a critical aspect of the death process.

Ferroptosis pathways appear to navigate lipids to unique shapes that promote the described changes. Whereas typical phospholipids are roughly cylindrical in shape – with head groups and tail groups that are of the same size – ferroptotic-competent lipids are conical, with PE head groups that are the smallest of the phospholipid headgroups, and wide tails due to the increased length, unsaturation, and incorporation of peroxides. These cones cannot not pack evenly in a bilayer, and thus would cause severe membrane disruptions, especially as peroxidation proceeds.

This model assumes simpler membranes than are found in cells, which can have up to a thousand lipid species in a membrane along with many proteins and complex inner and outer environments. Our simplifying assumption helps narrow in on the hypothesized mechanism, but could overlook other essential processes that are present during ferroptosis. Observed features that are not explained in this model will need to be considered, and future models will need to be updated to increase their explanatory power.

Furthermore, the measurements used to analyze this model are still in development, and will require more research to characterize the unique membrane properties that might be at play during ferroptosis. We used a measure of membrane width, area per lipid, membrane curvature, and membrane diffusion as indicators of what could lead to cell death. They paint an intuitive picture of how ferroptotic membranes might dysfunction, but they are not specific measures of death. A more complete measure of cell death in membranes might include aspects of these current measures, as well as other measures.

There have been several other molecular dynamics studies of oxidized lipid bilayers that can also shed light on the biophysical mechanisms underlying ferroptosis’^12–20^. Of these, most efforts were applied in united-atom models where all heavy atoms are modeled, and thus only allow for shorter spatial and temporal scales. Some lipid oxidation studies have also been recently done in coarse grained models, which have derived bilayer elastic coefficients (compressibility and bending) for oxidized membranes^15^, and the effect of cholesterol on protecting oxidized membranes^18^.

Atomistic molecular dynamics simulations consisting of 1-palmitoyl-2-linoleoyl-*sn*-glycero-3-phosphatidylcholine (PLPC) and its peroxidation and aldehyde products^16^ were reported to show that defects occurred with both aldehyde and peroxide lipids, but full pore formation only occurred for aldehyde lipids. At low concentrations of the aldehydes, these pores were stable, but at high concentrations the pores destabilized and micellization occurred. A related united-atom MD model showed that the addition of cholesterol can alleviate this destruction^13,18^. The effectiveness of aldehyde lipids in causing such destruction was hypothesized to result from their shorter and more mobile tails; peroxide lipids might be stabilized by hydrogen bonds with interfacial water. These aldehyde lipid products have not been previously observed in ferroptosis and provide a hypothesis to test experimentally.

Another united-atom study showed that peroxidized lipids lead to an important conformational change in membranes^14^. In simulations, the oxidized tails bend towards the water phase, with oxygen atoms forming hydrogen bonds with the water and with the polar lipid head group, leading to water defects and potential increase permeability to numerous substances.

Coarse-grained molecular dynamics has demonstrated the formation of micro-domains in membrane compositions consisting of saturated PC, unsaturated PC, and cholesterol^21^. Such clustering could imply that ferroptotic lipids aggregate locally, and the runaway feedback process described above would happen in concentrated micro-domains.

Future work on MD models of ferroptotic membranes could include investigating specific organelle membrane compositions – as has previously been done for the plasma membrane^22^ – to determine which organelles are more likely to be the cause of ferroptotic death. Such simulations could help explain the specific morphological changes of two key organelles during ferroptosis – mitochondria and endoplasmic reticulum (ER). In ferroptosis, mitochondria become shrunken, with membranes that are much denser, electron-dense, with reduced cristae, and outer membrane rupture^23^. ER compartments show highly organized oxygenation centers on one class of PE, and is specific toward arachidonic and adrenic FA tails^6^.

Finally, modeling and experimental data go hand-in-hand. Experimental data on lipid compositions are used for developing membrane models, and systematic modeling of membranes allow for generating hypotheses, which can be tested experimentally. We have generated here for the first time a hypothesis that during ferroptosis, which involves accumulation of oxidized-PUFA-containing phospholipids, membrane thinning and increased curvature drives increased accessibility to oxidants and ultimately micellization, resulting in irreversible damage to membrane integrity, which was verified in *in vitro* GUVs undergoing lipid peroxidation caused by photobleaching. This hypothesis can further guide biophysical and biochemical experiments on the execution mechanism of ferroptosis in the future.

## Methods

### Ferroptosis lipid composition

This study makes the simplifying assumption that the altered lipid composition observed in ferroptosis leads to membrane properties that can explain the type of cell death observed in ferroptosis. This altered composition includes increased concentrations of PUFAs, increased concentrations of PEs, increased concentration of lipid peroxides, and longer acyl tails. Similarly, altered lipid compositions are simulated in coarse-grained molecular dynamics to examine how they affect membrane properties.

### Peroxide beads

These membranes models were developed with a MARTINI force field, with added peroxide beads. The peroxide bead was identified with a systematic investigation of many different parameter values, and independently came upon parameters that agree with a those reported in^15^. This uses the original MARTINI model, with a 5-bead oleoyl, rather than the more recent 4-bead version. The optimal choice for the peroxide was found to be a combination of a hydrophobic MARTINI C3 bead for the unsaturated C=C bond, connected to a polar MARTINI P2 bead for the hydrophilic OOH. These beads are separated by 0.33 nm, smaller than the standard lipid bonding distance used in MARTINI (0.47 nm), and there is no defined angle between them, allowing the bead to move around freely.

### Membrane construction and simulation

The experiments used an algorithm that took as input the desired lipid scan, increments for the scan, and number of simulations for each given composition (10 times for all experiments shown here), membrane size, simulation duration, and as output it provided the final structure and trajectory of the simulated membrane from its initial to final configuration. The algorithm used a program called *insane*^24^ to generate model membranes according to membrane dimensions and relative lipid compositions, and solvate them – the models here solvated only in water. *Insane* (short for INSert membrANE) uses preset templates for orienting and placing lipid species into bilayer configurations, with relative lipid compositions and membrane size as inputs.

Lipids for these membranes are defined by the coarse-grained MARTINI force field^7^, which specify unique bead types for groups of 2, 3, or 4 heavy atoms. With this bead-specification, PE headgroups are modeled by the Qd (charged H-donor) bead, PC headgroups by the Q0 (charged non-bonding) bead, phosphate with Qa (charge H-acceptor) bead, glycerol with Na (nonpolar H-acceptor), groups of three saturated carbons with C1 (apolar), and groups of carbons with a double bond by a C3 (apolar, but slightly more than C1).

With a membrane of MARTINI lipids specified and solvated in water, the algorithm first runs energy minimization steps with *GROMACS*^25^, then a position-restrained *NVT* equilibration, a position-restrained *NpT* equilibration, an unrestrained NpT equilibration, and finally a production run in which the equilibrated membrane is simulated for the specified duration (10 µs for all simulations shown here).

### Measuring membrane properties

Area per lipid is a simple measurement, which takes the product of the *x* and *y* dimensions, and divides by the number of lipids in a single membrane leaflet. Width is measured by taking the difference between the average *z* position of the head groups in the two leaflets. Lipid diffusion was measured with the *GROMACS* function *gmx msd*, which computes the mean square displacement of molecules from their initial position. This was done for all lipid species present in a membrane, and a weighted average, with weights being the relative lipid compositions, gave the final lipid diffusion value. Curvature is calculated by interpolating two surfaces for the head groups of the two different leaflets, and measuring the numerical gradient of those surfaces–corresponding to the partial derivative of the *z* dimension over the *x*, *y* horizontal plane. The final curvature value is the average absolute value of the gradient.

#### Photobleaching of vesicles

Giant Unilamellar Vesicles (GUVs) were prepared by the classical electroformation method^26^. They contained 95% DOPC (with one unsaturation per chain) and 5% C5-Bodipy 530–550HPC. This lipid bears the fluorophore on the chain. Fluorescence excitation via epifluorescence with an Argon laser λ = 514 nm) leads to bleaching of the fluorophore. The relative GUV surface change upon photobleaching was measured using micropipette aspiration^27^. It can be deduced from the length of the tongue over time, at constant aspiration pressure.
